# Successful use of anti-CD19 CAR T cells in severe treatment-refractory stiff-person syndrome

**DOI:** 10.1073/pnas.2403227121

**Published:** 2024-06-17

**Authors:** Simon Faissner, Jeremias Motte, Melissa Sgodzai, Christian Geis, Aiden Haghikia, Dimitrios Mougiakakos, Dominic Borie, Roland Schroers, Ralf Gold

**Affiliations:** ^a^Department of Neurology, Ruhr-University Bochum, St. Josef-Hospital, Bochum 44791, Germany; ^b^Department of Neurology, Jena University Hospital, Jena 07747, Germany; ^c^Department of Neurology, Medical Faculty, Otto-von-Guericke University, Magdeburg 39120, Germany; ^d^Department of Hematology, Medical Faculty, Otto-von-Guericke University, Magdeburg 39120, Germany; ^e^Kyverna Therapeutics, Emeryville, CA 94608; ^f^Department of Haematology and Oncology, Ruhr-University Bochum, Knappschaftskrankenhaus Bochum, Bochum 44892, Germany

**Keywords:** CAR T cells, chimeric antigen receptor, stiff-person syndrome, neuroimmunological disorder, B cell autoimmunity

## Abstract

Treatment with autologous chimeric antigen receptor (CAR) T cells has emerged as a highly effective approach in neuroimmunological disorders such as myasthenia gravis. We report a case of successful anti-CD19 CAR T cell use in treatment-refractory stiff-person syndrome (SPS). To investigate clinical and immunological effects of anti-CD19 CAR T cell use in treatment-refractory SPS, a 69-y-old female with a 9-y history of treatment-refractory SPS with deteriorating episodes of stiffness received an infusion of autologous anti-CD19 CAR T cells (KYV-101) and was monitored clinically and immunologically for more than 6 mo. CAR T cell infusion resulted in reduced leg stiffness, drastic improvement in gait, walking speed increase over 100%, and daily walking distance improvement from less than 50 m to over 6 km within 3 mo. GABAergic medication (benzodiazepines) was reduced by 40%. KYV-101 CAR T cells were well tolerated with only low-grade cytokine release syndrome. This report of successful use of anti-CD19 CAR T cells in treatment-refractory SPS supports continued exploration of this approach in SPS and other B cell–related autoimmune disorders.

## Background

Stiff-person syndrome (SPS) is characterized by progressive rigidity and muscle spasms, which usually affect axial and limb muscles (1). SPS can be classified into classic SPS or variants such as focal or segmental SPS, among others (1). In most patients, antibodies against amphiphysin or glutamic acid decarboxylase (GAD) can be detected (1). The antineuronal immunopathology including autoantibodies and cellular mechanisms specifically targeting GABAergic inhibitory pathways and synaptic signaling machinery are believed to contribute to pathogenesis (2, 3). SPS associated with antibodies against amphiphysin is also often accompanied by the occurrence of neoplastic disease (1).

Treatment approaches targeting B cell–driven pathogenic mechanisms such as plasma exchange, intravenous immunoglobulin, anti-CD20-directed approaches, or immunosuppressants have met variable success (4). Anti-CD19 chimeric antigen receptor (CAR) T cells were originally developed for the treatment of B cell hematologic malignancies and have recently emerged as a promising approach for autoimmune disorders. This report adds to the emerging clinical experience on the use of anti-CD19 CAR T cells in neuroimmunological disorders affecting the central nervous system (CNS) (5).

## Methods

**Fig. 1.**
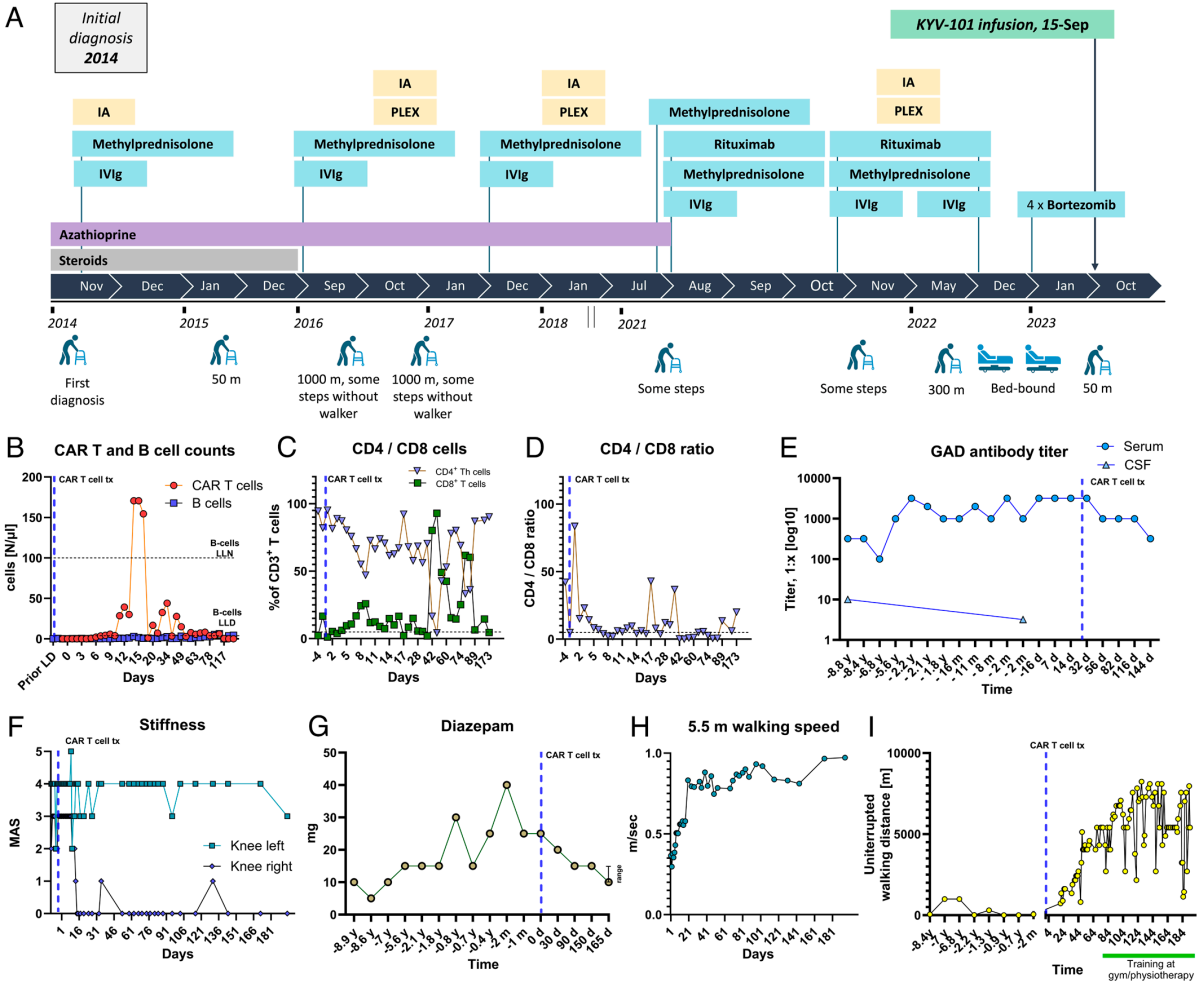
Immunological and clinical findings in a patient with treatment-refractory SPS, successfully treated with anti-CD19 CAR T cells. Panel (*A*) shows the clinical course of the patient over a period of 9 y following initial diagnosis. Panel (*B*) shows CAR T cell and CD19^+^ B cell counts, measured using flow cytometry. Panel (*C*) shows CD4^+^ and CD8^+^ T cells as percent of CD3^+^ T cells, and panel (*D*) shows the CD4/CD8 ratio. Panel (*E*) depicts the development of anti-GAD65 titers in serum and cerebrospinal fluid over the disease course. Panel (*F*) shows leg stiffness following administration of CAR T cells using the modified Ashworth scale (MAS). Panel (*G*) shows daily diazepam dosage as symptomatic GABAergic treatment of stiffness. Panel (*H*) shows the mean of two measurements for the 5.5 m short distance walking speed in m/s following treatment. Panel (*I*) shows daily walking distance using a wheeled walker before and after CAR T cells. Abbreviations: CAR = chimeric antigen receptor; d = day; GAD = glutamic acid decarboxylase; LLD = lower limit of detection; LLM = lower limit of normal; m = month; tx = treatment; y = year.

The female patient first presented with symptoms at the age of 59 in 2014. She reported sudden loss of leg control, leading to repeated falls. Workup detected anti-GAD65 immunoglobulin (Ig)G both in the cerebrospinal fluid (CSF) (titer 1:10) and serum (titer 1:320). Immunotherapy led to disease stabilization for 3 y with subsequent decrease of walking distance needing wheeled walker support despite escalation to rituximab and bortezomib (Fig. 1*A*; *SI Appendix* for extended patient history). The therapeutic strategy targeting B-cells was escalated to use of a second-generation anti-CD19 CAR T cell approach (KYV-101, Kyverna Therapeutics, Inc., CA) (6) (*SI Appendix* for extended methods). The patient provided written informed consent for treatment and publication. Biosampling of whole blood and serum was approved by Ruhr-University Bochum (20-6827), the use of the CAR T cell approach received approval by state authorities (district government Arnsberg, 24.05.09-012/2023-001). Under German law, individualized treatment approaches can be made in the case of severe, potentially life-threatening diseases.

## Results

### Treatment with Anti-CD19 CAR T Cells.

Autologous T cells were collected from the patient’s peripheral blood and transduced with KYV-101. Following successful in vitro expansion and lymphodepletion with fludarabine (30 mg/m^2^; days −6, −5, and −4) and cyclophosphamide (300 mg/m^2^; days −6, −5, and −4), the patient received a single infusion of 1 × 10^8^ anti-CD19 CAR T cells (day 0). CAR T cells expanded beginning on day +5 to a maximum of 56.7% of all CD3^+^ T cells at day +14 (171 cells/µL). B cells remained low (Fig. 1*B*). Frequencies of CD8^+^/CD3^+^ T cells increased until onset of CAR T cell expansion with subsequent decline. Thereafter, CD8^+^/CD3^+^ T cells increased again from day 42 onward, resulting in an altered CD4/CD8 ratio (Fig. 1 *C*/*D*).

### Safety.

Following CAR T cell administration, the patient developed fever (maximum of 38.3 °C) and transient hypotension corresponding to grade 2 cytokine release syndrome (CRS) by day +9, successfully treated with paracetamol, dexamethasone, and tocilizumab. Indicative of tissue-based expansion of anti-CD19 CAR T cells, concurrent sore throat and cervical lymph node swelling were observed on day +9 and promptly resolved upon CRS treatment. Transient and limited (~4-fold) increases in liver transaminases (maximum at day +15) were spontaneously reversible (day +45).

### Immunological and Clinical Improvements.

Following administration of anti-CD19 CAR T cells, anti-GAD65 titers decreased from 1:3,200 at baseline to 1:1,000 at day +56 and to 1:320 by day +144 (Fig. 1*E*). Clinically, there was marked improvement of stiffness. The Modified Ashworth scale (MAS) score for the right knee decreased from 2 to 3 at baseline to 0 beginning at day +14. MAS score for the left knee fluctuated around 4 and improved modestly over time (Fig. 1*F*). Pain improved from a numeric rating scale (NRS) score of 4 at baseline to several assessments of an NRS score of 0 beginning at day +50. GABAergic medication could be reduced stepwise from 25 to 10 to 15 mg within 5 mo (Fig. 1*G*). Fatigue, assessed using the fatigue severity scale improved modestly from 48 points prior to CAR T cells to 40 points at day +112. Most impressively, walking ability improved substantially. A 5.5-m walking test using a wheeled walker demonstrated a walking speed increase of more than 100% from approximately 0.37 m/s at day +1 to 0.83 m/s at day +20 (Fig. 1*H* and Movie S1). Uninterrupted walking distance at home increased from several meters at baseline to more than 4 km after day 50 and more than 6 km after day 90 (Fig. 1*I*). Importantly, no further immunotherapy such as IVIg was administered post-CAR T cell infusion.

## Discussion

We describe a case of successful use of anti-CD19 CAR T cells in treatment-refractory SPS. B cell–directed therapy with anti-CD19 CAR T cells has recently emerged as a promising approach for the treatment of a variety of autoimmune disorders. Efficacy was first demonstrated in five patients with systemic lupus erythematosus with drug-free remission ongoing after a median follow-up of 8 mo (7). Additionally, in patients with anti-synthetase syndrome (8, 9), systemic sclerosis (10, 11), and myasthenia gravis (MG) (12), robust efficacy was observed. These findings add to the recent report of positive effects of CAR T cell approaches in a nonrandomized phase 1b/2a study in MG assessing the impact of T cells transduced with RNA (rCAR-T) targeting B cell maturation antigen (BCMA) (13). While recognizing that quantification of anti-acetylcholine receptor antibodies and anti-GAD65 antibodies uses different methodologies, anti-GAD65 titers declined within 56 d from 1:3,200 to 1:1,000, and to 1:320 after 5 mo, with dynamics similar to those reported in MG for anti-acetylcholine receptor antibodies (12). Assessment of changes of anti-GAD65 antibodies in CSF could have been more informative and should be considered in clinical trials. These findings demonstrate that CD19-expressing plasmablasts and short-lived plasma cells are susceptible to depletion by anti-CD19 CAR T cells (12, 14). The reduction of anti-GAD65 titers may have contributed to the observed clinical improvement, even if a direct pathogenic role for these autoantibodies in SPS is still debated (15). The recently reported presence of KYV-101 anti-CD19 CAR T cells in the CSF of patients with multiple sclerosis and the associated impact on oligoclonal bands (5) may have contributed to SPS disease improvement given the demonstrated role of clonal GAD-specific B cell activation in the CNS (16), and the accompanying intrathecal synthesis of anti-GAD autoantibodies (17). This may also have contributed to reduced T cell priming, potentially involved in GAD65-directed autoimmunity.

Until now, it remains unclear how CAR T cell treatment might affect the repopulation of B cells or the niche of CD19^neg^ long-lived plasma cells. We hypothesize that the deep depletion of B cells might favor the repopulation of a naive B cell phenotype, which should be investigated in further studies. The patient reported here displayed only modest improvement of stiffness, likely due to the long-lasting disease course. Autopsy of a patient with severe anti-GAD65 SPS with a >18-mo disease course, nonresponsive to multiple immunotherapies, documented spinal degeneration due to neuronal loss associated with microgliosis, which may explain residual stiffness here (18). Moreover, stiffness was assessed using the MAS to document potentially subtle changes. Given its validation and precedented use in clinical trials, future clinical trials should consider the use of the stiffness index (19). To address this possible limitation, in clinical trials, the patients’ clinical assessment could be complemented by the addition of an electrophysiological assessment of the degree of reciprocal inhibition (20).

Importantly, consistent with emerging experience regarding the use of anti-CD19 CAR T cells for the treatment of autoimmune diseases (7–11) including MG (12), no severe safety signals such as CRS grade ≥ 3 or immune effector cell associated neurotoxicity syndrome grade ≥ 3 were observed. Acceptable tolerability of KYV-101 may be related to the lower level of cytokines being produced in response to engagement of the fully human-derived CAR (21), which translated into a 10-fold reduction of severe neurotoxic events in patients with B cell lymphoma (6). The 6-mo follow-up of the patient presented here is still limited. Further follow-up, as recently reported for a series of 15 autoimmune rheumatology patients treated with anti-CD19 CAR T cells (22), will help assess the potential in neuroimmunological diseases.

These results provide additional evidence supporting the further evaluation through controlled clinical trials of the use of anti-CD19 CAR T cells as a potentially effective therapeutic approach in refractory B cell–related neuroimmunological disorders.

## Consent for Publication.

The patient provided written informed consent for the publication of the case and the video material.

## Supplementary Material

Appendix 01 (PDF)

Movie S1. **Video documentation of a patient with treatment-refractory Stiff person syndrome, successfully treated with anti-CD19 Chimeric Antigen Receptor (CAR) T Cells.** Walking two days prior, four months and 6.5 months after CAR T-cell therapy, depicting marked improvement of stiffness, gait and walking speed using a wheeled walker.

## Data Availability

All study data are included in the article and/or supporting information.
