# Supplementary material for: Successful use of anti-CD19 CAR T cells in severe treatment-refractory stiff-person syndrome
Source: Proc Natl Acad Sci U S A. 2024 Jun 17;121(26):e2403227121. doi: 10.1073/pnas.2403227121 (PMC11214089; doi:10.1073/pnas.2403227121)
Supplement: Supplementary file 1 — Appendix 01 (PDF) [file pnas.2403227121.sapp.pdf]

# Successful use of anti-CD19 CAR T-cells in severe treatment-refractory stiff-person-syndrome

Simon Faissner, M.D. [1*#], Jeremias Motte, M.D. [1*], Melissa Sgodzai. M.Sc. [1*], Christian Geis, M.D. [2], Aiden Haghikia, M.D. [3], Dimitrios Mougiakakos, M.D.[4], Dominic Borie, M.D., Ph.D.[5], Roland Schroers, M.D. [6#], Ralf Gold, M.D. [1#]

[1] Department of Neurology, Ruhr-University Bochum, St. Josef-Hospital, Bochum, Germany

[2] Department of Neurology, Jena University Hospital, Am Klinikum 1, 07747, Jena, Germany.

[3] Department of Neurology, Medical Faculty, Otto-von-Guericke University, Magdeburg, Germany

[4] Department of Hematology, Medical Faculty, Otto-von-Guericke University, Magdeburg, Germany

[5] Kyverna Therapeutics, Emeryville, CA, USA.

[6] Department of Haematology and Oncology, Ruhr-University Bochum, Knappschaftskrankenhaus Bochum, Germany.

* These authors contributed equally as shared first authors

# Corresponding authors

Prof. Ralf Gold, MD, Prof. Simon Faissner, MD,
Department of Neurology
Ruhr-University Bochum, St. Josef-Hospital
Gudrunstr. 56, 44791 Bochum, Germany
Tel: +49-234-5092411; Fax: +49-234-5092740
Email: ralf.gold@rub.de; simon.faissner@rub.de;

Prof. Roland Schroers, MD
Department of Hematology
Ruhr-University Bochum
In der Schornau 23/25
44892 Bochum, Germany
roland.schroers@rub.de

# SI APPENDIX

## Methods

### Patient History

The female patient first presented with symptoms at the age of 59 in 2014. She reported sudden loss of leg control, leading to repeated falls. Work-up detected anti-GAD65 immunoglobulin (Ig)G both in the cerebrospinal fluid (CSF) (titer 1:10) and serum (titer 1:320). Onconeuronal antibodies, including anti-glycine receptor antibodies, and a diagnostic work-up including PET-CT for malignancies, were negative. Treatment with corticosteroids and intravenous Ig (IVIg) resulted in only minor improvement of rigidity, and therefore a series of plasma exchanges was performed. Treatment with azathioprine, prednisolone, and diazepam (Figure 1a) led to disease stabilization for three years. Subsequent exacerbations were treated with a combination of plasma exchanges and immunoadsorptions. The patient's walking distance, however, decreased progressively to 500 meters and she required wheeled walker support. In 2021, an increase of serum anti-GAD65 antibody-titers (1:3200) associated with gait imbalance that restricted walking ability to a few steps, was observed. Immunotherapy was escalated to rituximab (three courses), but severe leg stiffness remained and resulted in a bed-bound state. In 2023, therapy was escalated using the proteasome inhibitor bortezomib with only transient stabilization. B-cell targeting therapeutic strategy was escalated to use of an anti-CD19 CAR T-cell approach.

### Anti-CD19 CAR Construct and Evaluations

CAR T-cells were engineered using a second generation anti-CD19 CAR construct (KYV-101, Kyverna Therapeutics, Inc. CA, USA) comprising a fully human CD19 binding domain, a CD8α hinge and transmembrane domain, a CD28 co-stimulatory domain, and a CD3ζ activation domain (1). Clinical evaluations were performed at regular intervals following administration of KYV-101. Stiffness was assessed using the Modified Ashworth scale (MAS). Pain was assessed using the numeric rating scale (NRS, range 0-10). Fatigue was assessed using the fatigue severity scale (range 9-63). Anti-GAD65 titers were quantified using an immunofluorescence test of transfected cells (Euroimmun, Germany).

## References

1. J. N. Brudno *et al.*, Safety and feasibility of anti-CD19 CAR T cells with fully human binding domains in patients with B-cell lymphoma. *Nature medicine* **26**, 270-280 (2020).

## Video 1

**Video documentation of a patient with treatment-refractory Stiff person syndrome, successfully treated with anti-CD19 Chimeric Antigen Receptor (CAR) T Cells.** Walking two days prior, four months and 6.5 months after CAR T-cell therapy, depicting marked improvement of stiffness, gait and walking speed using a wheeled walker.
